# Editorial: Adenosine metabolism in cardiovascular and smooth muscle

**DOI:** 10.3389/fphar.2026.1829428

**Published:** 2026-03-24

**Authors:** Barbara Kutryb-Zając, Simon C. Robson, Elisabetta Caiazzo

**Affiliations:** 1 Department of Biochemistry, Medical University of Gdansk, Gdańsk, Poland; 2 Centre of Experimental Cardiooncology, Medical University of Gdansk, Gdańsk, Poland; 3 Departments of Anesthesiology and Medicine, Center for Inflammation Research, Beth Israel Deaconess Medical Center and Harvard Medical School, Boston, MA, United States; 4 Department of Pharmacy, School of Medicine and Surgery, University of Naples “Federico II”, Naples, Italy; 5 School of Infection and Immunity, College of Medical, Veterinary and Life Sciences, University of Glasgow, Glasgow, United Kingdom

**Keywords:** adenosine deaminase, adenosine metabolism, cardiovascular inflammation, CD73, ecto-nucleotidases, endothelium, heart failure with preserved ejection fraction (HFpEF)

Adenosine and its metabolic pathways represent a foundational signaling system in cardiovascular physiology, integrating metabolic stress, inflammation, and vascular homeostasis (Burnstock, 2017; Caiazzo et al., 2020; Antonioli et al., 2023). By regulating vascular tone, cardiac function, and immune responses, adenosine has emerged as a critical nexus between cellular energetics and systemic cardiovascular health (Eltzschig et al., 2012; Headrick et al., 2013). Disruptions in adenosine metabolism and signaling are implicated in hypertension, heart failure, vascular remodeling, and septic cardiovascular dysfunction (Gaudry et al., 2020; Kutryb-Zając et al., 2023; Vergadi et al., 2020; Marchi et al., 2024), making this pathway a fertile ground for pharmacological innovation.

In particular, the coordinated activity of vascular ecto-nucleotidases, such as CD39 and CD73, critically regulates extracellular adenosine availability and inflammatory balance (Robson et al., 2006). This Research Topic brings together a collection of experimental and translational studies that illuminate both mechanistic insights and therapeutic potential of modulating adenosine-related pathways in cardiovascular and smooth muscle contexts.

This Research Topic includes five original research articles and one review, each addressing distinct facets of extracellular adenosine biology, from enzymatic activity and receptor signaling to translational targets in disease and experimental models. The review by Lunderberg et al. emphasizes CD73 as a druggable target, where the conversion of AMP to adenosine protects against vascular and systemic inflammation. Strategies such as CD73 induction, supplementation, or combined CD39/CD73 modulation may enhance adenosine-mediated signaling, improving tolerance to ischemia and inflammatory stress. Early clinical data suggest that increased CD73 activity correlates with decreased vascular leakage, less cytokine-mediated damage, and improved outcomes in critically ill patients. These findings position CD73 modulation as a promising approach to restore purinergic balance in severe cardiovascular disease and in other inflammatory conditions.

On the enzymatic front, Skaldin et al. describe the development of anti-ADA2 single-chain antibodies coupled to alkaline phosphatase for diagnosing pleural tuberculosis. While primarily focused on infectious immunology, the study introduces highly specific tools for detecting ADA2, a key regulator of extracellular adenosine metabolism and immune signaling. Such tools may facilitate translational applications in cardiovascular disorders where inflammation and purinergic dysregulation intersect.

Complementing this, Kawecka et al. investigate how adenosine deaminase mediates endothelial inflammation via an ADA1-CD26 interaction in post-COVID vascular injury. Chronic vascular sequelae following SARS-CoV-2 infection remain a major clinical concern, and this study shows that ADA1 binds to CD26, competing with the virus and temporarily limiting viral entry, but post-infection this interaction promotes long-term vascular complications. Exposure of endothelial cells to post-COVID sera increased ADA1 and CD26 expression, induced a pro-inflammatory phenotype, and enhanced monocyte/macrophage adhesion. These effects reflect both ADA1 enzymatic degradation of adenosine and its non-enzymatic adhesive function. Recombinant inhibitors of ADA1-CD26 binding reduced immune cell adhesion, highlighting the functional relevance of this interaction.

In the context of vascular remodeling, Sun and Du demonstrate that the AMPK activator O304 limits abdominal aortic aneurysm (AAA) progression by preventing vascular smooth muscle cell (VSMC) phenotypic switching from the contractile to the synthetic state. While not directly targeting adenosine receptors, AMPK engagement influences nucleotide metabolism and vascular integrity. *In vivo*, O304 inhibited aneurysm expansion and lowered systolic and diastolic blood pressure, highlighting both structural and systemic protective effects. Unlike indirect AMPK activators such as metformin, O304 maintains AMPK activity without altering cellular energy balance, illustrating a targeted strategy to modulate VSMC behavior and extracellular remodeling in AAA.

The central nervous system modulation of cardiovascular defense mechanisms is addressed by El-Naggar et al., who show that central adenosine A_3_ receptors suppress cholinergic protection in sepsis, implicating PI3K/MAPKs/NFκB signaling. Adenosine A_3_ receptors exacerbate autonomic dysfunction by dampening protective cholinergic reflexes. These findings suggest that selective targeting of central purinergic receptors could restore cardiovascular stability in septic states.

Riemma et al. investigate adenosine pathway alterations in heart failure with preserved ejection fraction (HFpEF) using a salt-sensitive rat model. The study reveals broad dysregulation of adenosine metabolism and transport, with reductions in CD39, CD73, ADA, and nucleoside transporters, alongside altered receptor expression: increased A_1_ and shifts in A_2A_, A_2B_, and A_3_. These molecular changes associate with diastolic dysfunction, microvascular impairment, fibrosis, and oxidative stress. Notably, selective activation of A_2A_ receptors *in vitro* attenuated profibrotic gene expression and restored antioxidant defenses in endothelial cells, supporting receptor-specific approaches to restore vascular homeostasis and suggesting potential therapeutic strategies for HFpEF.

Collectively, these contributions underscore key themes in adenosine biology. First, enzymes such as CD73, ADA1, and ADA2 act as active regulators of inflammation and vascular signaling rather than passive metabolic catalysts. Second, adenosine receptor diversity mediates context-dependent effects, from metabolic adaptation to autonomic regulation. Third, purinergic signaling intersects with broader stress-response pathways, including AMPK and NFκB cascades, emphasizing the need for systems-level perspectives in therapeutic design.

Translationally, these studies suggest that precise modulation of adenosine metabolism through enzyme targeting, receptor biasing, or metabolic adaptation may address unmet clinical needs in vascular inflammation, aneurysmal disease, sepsis-associated cardiovascular dysfunction, and HFpEF. Integration of biochemical diagnostics (e.g., ADA2 profiling), receptor-specific ligands, and metabolic modulators may enable tailored interventions that restore purinergic equilibrium without systemic side effects.

In summary, advancing our understanding of adenosine pathways in cardiovascular and smooth muscle systems reveals a complex, dynamic network of enzymatic, receptor, and metabolic controls. Papers presented in this Research Topic highlights the therapeutic potential of modulating these pathways and lay a foundation for future translational efforts to harness purinergic pharmacology for cardiovascular health.

## References

[B1] Antonioli L. Fornai M. Pellegrini C. Pacher P. Haskó G. (2023). Adenosine signaling as target in cardiovascular pharmacology. Curr. Opin. Pharmacol. 71, 102393. 10.1016/j.coph.2023.102393 37450948 PMC10527223

[B2] Burnstock G. (2017). Purinergic signaling in the cardiovascular system. Circ. Res. 120, 207–228. 10.1161/CIRCRESAHA.116.309726 28057794

[B3] Caiazzo E. Cerqua I. Riemma M. A. Turiello R. Ialenti A. Schrader J. (2020). Exacerbation of allergic airway inflammation in mice lacking ecto-5′-nucleotidase (CD73). Front. Pharmacol. 11, 589343. 10.3389/fphar.2020.589343 33328996 PMC7734328

[B4] Eltzschig H. K. Sitkovsky M. V. Robson S. C. (2012). Purinergic signaling during inflammation. N. Engl. J. Med. 367 (24), 2322–2333. 10.1056/NEJMra1205750 23234515 PMC3675791

[B5] Gaudry M. Vairo D. Marlinge M. Gaubert M. Guiol C. Mottola G. (2020). Adenosine and its receptors: an expected tool for the diagnosis and treatment of coronary artery and ischemic heart diseases. Int. J. Mol. Sci. 21 (15), 5321. 10.3390/ijms21155321 32727116 PMC7432452

[B6] Headrick J. P. Ashton K. J. Rose’Meyer R. B. Peart J. N. (2013). Cardiovascular adenosine receptors: expression, actions and interactions. Pharmacol. Ther. 140, 92–111. 10.1016/j.pharmthera.2013.06.002 23764371

[B7] Kutryb-Zając B. Kawecka A. Nasadiuk K. Braczko A. Stawarska K. Caiazzo E. (2023). Drugs targeting adenosine signaling pathways: a current view. Biomed. Pharmacother. 165, 115184. 10.1016/j.biopha.2023.115184 37506580

[B8] Marchi E. Muraca I. Berteotti M. Gori A. M. Valenti R. Marcucci R. (2024). Adenosine in interventional cardiology: physiopathologic and pharmacologic effects in coronary artery disease. Int. J. Mol. Sci. 25 (11), 5852. 10.3390/ijms25115852 38892037 PMC11172110

[B9] Robson S. C. Sévigny J. Zimmermann H. (2006). The E-NTPDase family of ectonucleotidases: structure, function and pathophysiological significance. Purinergic Signal 2, 409–430. 10.1007/s11302-006-9003-5 18404480 PMC2254478

[B10] Vergadi E. Vapaatalo H. Kytö V. (2020). Adenosine in cardiovascular inflammation and remodeling. Int. J. Mol. Sci. 21, 8060. 10.3390/ijms21218060 33137927 PMC7663462

